# Further evidences of an emerging stingless bee-yeast symbiosis

**DOI:** 10.3389/fmicb.2023.1221724

**Published:** 2023-08-11

**Authors:** Gabriela Toninato de Paula, Weilan Gomes da Paixão Melo, Ivan de Castro, Cristiano Menezes, Camila Raquel Paludo, Carlos Augusto Rosa, Mônica Tallarico Pupo

**Affiliations:** ^1^Department of Pharmaceutical Sciences, School of Pharmaceutical Sciences of Ribeirão Preto, University of São Paulo, Ribeirão Preto, Brazil; ^2^Center for Agricultural and Natural Sciences and Letters, State University of the Tocantina Region of Maranhão, Estreito, Brazil; ^3^Department of Genetics, Faculty of Medicine of Ribeirão Preto, University of São Paulo, Ribeirão Preto, Brazil; ^4^Brazilian Agricultural Research Corporation, Jaguariúna, Brazil; ^5^Institute of Biological and Health Sciences, Federal University of Mato Grosso, Barra do Garças, Brazil; ^6^Departamento de Microbiologia, Instituto de Ciências Biológicas, Universidade Federal de Minas Gerais, Belo Horizonte, Brazil

**Keywords:** stingless bees, fungi, yeast, *Zygosaccharomyces*, symbiosis, brood cell

## Abstract

Symbiotic interactions between microorganisms and social insects have been described as crucial for the maintenance of these multitrophic systems, as observed for the stingless bee *Scaptotrigona depilis* and the yeast *Zygosaccharomyces* sp. SDBC30G1. The larvae of *S. depilis* ingest fungal filaments of *Zygosaccharomyces* sp. SDBC30G1 to obtain ergosterol, which is the precursor for the biosynthesis of ecdysteroids that modulate insect metamorphosis. In this work, we find a similar insect-microbe interaction in other species of stingless bees. We analyzed brood cell samples from 19 species of stingless bees collected in Brazil. The osmophilic yeast *Zygosaccharomyces* spp. was isolated from eight bee species, namely *Scaptotrigona bipunctata*, *S. postica*, *S. tubiba*, *Tetragona clavipes*, *Melipona quadrifasciata*, *M. fasciculata*, *M. bicolor*, and *Partamona helleri*. These yeasts form pseudohyphae and also accumulate ergosterol in lipid droplets, similar to the pattern observed for *S. depilis*. The phylogenetic analyses including various *Zygosaccharomyces* revealed that strains isolated from the brood cells formed a branch separated from the previously described *Zygosaccharomyces* species, suggesting that they are new species of this genus and reinforcing the symbiotic interaction with the host insects.

## 1. Introduction

Insects establish several symbiotic interactions with microorganisms, ranging from obligate mutualisms to specialized parasitism (Van Arnam et al., 2018; Menegatti et al., 2021). Because insects are unable to synthesize steroids, many vitamins, and enzymes to degrade plant cell wall materials, they end up relying on microbes, such as fungi, to perform these functions (Blackwell, 2017). Bacterial symbionts also play different roles in such multitrophic interactions, like production of chemical defenses against entomopathogens (Van Arnam et al., 2018; Menegatti et al., 2021).

The nutritional benefits provided by fungal mutualists is well described for fungus-growing ants, native to the Neotropics, and termites from Africa and Asia (Mueller et al., 2001). Ants of the subtribe Attina cultivate fungi of the families Agaricaceae and Pterulaceae for food (Bizarria et al., 2022). Similarly, termites of the subfamily Macrotermitinae cultivate fungi of the genus *Termitomyces*, which are not only the main food source but also provide digestive services (De Fine Licht et al., 2005).

Yeasts have been reported in association with insects. *Saccharomyces cerevisiae* is important for larval development of *Drosophila melanogaster* by providing nutrients such as organic nitrogen, essential vitamins, and lipids, and also mediating attraction to overripe fruits and in oviposition (Becher et al., 2012; Stefanini, 2018). Female ambrosia beetles carry some fungi, such as yeast, in mycangial pouches. After the eggs hatch, a yeast-like fungal growth develops to serve as food for the larvae. Yeast-like symbionts of beetles in the family Anobiidae, such as *Symbiotaphrina* species found in the insect gut, are passed from the female to the next generation by spreading yeast cells on her eggs, which are ingested by the larvae after they hatch from the eggs (Blackwell, 2017).

Stingless bees (SBs) (Apidae: Apinae: Meliponini) are a monophyletic group of eusocial insects that belong to a larger group of corbiculate bees (Romiguier et al., 2016). Meliponini have pantropical distribution and interact with various microorganisms such as bacteria, yeasts, filamentous fungi, and viruses. Indeed, microbial fermentation contributes to important physicochemical characteristics and to the preservation of pollen and honey (De Paula et al., 2021).

There has been a growing interest in studies on the microbiomes of SBs, including comparisons with honey bees. The benefits provided by the yeast *Zygosaccharomyces* sp. SDBC30G1 to the host *Scaptotrigona depilis* were reported as the first example of nutritional symbiosis in SBs. This osmophilic yeast grows inside the brood cells of *S. depilis* and is eaten by the larvae, an essential process for larval metamorphosis. The fungus accumulates ergosterol, which is used by the developing insect as a precursor for ecdysteroid biosynthesis, leading to the proper pupation (Menezes et al., 2015; Paludo et al., 2018). Other fungi isolated from the cerumen of *S. depilis* seem to control the development of *Zygosaccharomyces* SDBC30G1. *Candida* sp. SDCP2 produces volatile alcohols that stimulate the growth of *Zygosaccharomyces* sp. SDBC30G1. *Monascus ruber* SDBC1 negatively modulate the growth of both *Zygosaccharomyces* sp. SDBC30G1 and *Candida* sp. SDCP2 through the production of lovastatin and monascin, respectively (Paludo et al., 2019). More recently, Rosa-Fontana et al. (2022) reported the presence of fungivorous mites in brood cells of the *Scaptotrigona postica*, which might be involved in controlling the multiplication of both mutualistic and opportunistic fungi in the brood cell favoring the larval development of this SB.

Bacterial strains are also associated with bees. Cerqueira et al. (2021) reported that SBs of the genus *Melipona* lack the associated gut bacterial symbionts *Snodgrassella* and *Gilliamella*, important for the health of honey bees and bumblebees. To compensate this absence, *Melipona* bees would have as symbionts newly acquired microorganisms or persistent members of the ancestral microbiome. Interestingly, bacterial symbionts isolated from the brood cells and from the cuticle of *Melipona scutellaris* SBs were found to produce antimicrobial compounds that are likely involved in the production of antimicrobial defenses against entomopathogens (Menegatti et al., 2018, 2020; Rodriguez-Hernandez et al., 2019). *S. depilis* larvae also hold *Bacillus* sp. in their gut, which encodes biosynthetic gene clusters for the production of antimicrobial compounds (Paludo et al., 2016). Altogether, these findings suggest complex microbial interactions in SBs.

The unprecedented importance of *Zygosaccharomyces* for the larval development had been observed just for one SB species so far, lacking generality among Meliponini (Roubik, 2023), and raised the hypothesis that similar symbiosis might be more spread among SBs. Here, we describe the occurrence of *Zygosaccharomyces* isolates in the brood cells of different SBs species, as well as the phylogenetic relationships and morphological characteristics of these yeasts in comparison with other species isolated from other locations in the colonies of SBs.

## 2. Materials and methods

### 2.1. Insect collection and fungal isolation

Permits for collection of biological samples and research were issued by the Brazilian government (SISBIO 46555-6, CNPq 010936/2014-9, SisGen A25AA57, SisGen A9D808C). Samples of brood cells, honey and cerumen from the nests of different SBs species were collected from March 2018 to November 2019 at different sites in southeastern Brazil. The Global Positioning System (GPS) coordinates, information about the isolated microorganisms and the SBs species can be found in Supplementary Table 1 (Supplementary material). The growth of white microorganisms in brood cells was visualized in stereomicroscope, and the pseudohyphae were carefully collected with a pointed spatula and aseptically seeded on Petri dishes containing 30 G agar medium (30 g glucose, 3 g malt extract, 3 g yeast extract and 2 g agar on 100 mL deionized water, pH 6.0) (Paludo et al., 2018). Other parts of the colonies (honey, cerumen, and larval food) were also assessed for fungal isolation. One gram of each material was transferred to a 1.5 mL reaction tube containing 200 μL of the 30 G liquid medium (30 g glucose, 3 g malt extract, 3 g yeast extract, and 100 mL deionized water, pH 6.0). Samples were shaken for 30 s and transferred to Petri dishes containing 30 G agar medium. Petri dishes were incubated at 29°C until the beginning of microbial growth and serial cultures were established to obtain pure cultures. Stocks were stored in cryovials at −80°C in a homogenized mixture of 30 G liquid medium supplied with 20% glycerol.

### 2.2. Cell morphology

Images of a small portion of the microorganisms grown in a Petri dish and images directly obtained from the brood cells were registered by stereo microscope (Leica EZ4 W) integrated 5-megapixel cameras to analyze the phenotype of each strain. To analyze cell morphology, cells were harvested using a platinum loop and transferred to a slide containing 50 μL of saline (0.85% NaCl). The cells were mixed on the slide attached to this homogenate and then observed under an optical microscope (Leica ICC50 HD) at 40× magnification.

### 2.3. Analysis of steroids by fluorescence microscopy

Staining of cytoplasmic lipid-accumulating organelles, also known as adiposomes, was performed according to the methodology described by Hoiczyk et al. (2009) and the procedure previously standardized in our laboratory (Paludo et al., 2018). Cells fixed on glass slides at 40°C were stained with 50 μL of Nile Red dye [Nile Red, Sigma-Aldrich (St. Louis, MO, USA)] for 30 min and dissolved in ethanol (10 μg/mL) in the dark. After this time, cells were quickly washed to remove excess dye and observed under a fluorescence microscope. Analyses were performed using the Leica TCS SP5 confocal microscope.

### 2.4. DNA extraction and fungal identification

Genomic DNA was extracted following an adapted protocol of Sharma and Singh (2005). Fungi were grown in 30 G agar medium at 29°C until sufficient biomass was achieved. After growth, a portion of each culture was removed and transferred to 2 mL reaction tubes containing 500 μL TE buffer 0.01X (X: 6.06 g Tris-HCl, 1.86 g EDTA and 50 mL deionized water, pH 8.0), 3 μL RNase (7,000 units/mL) was added to digest the RNA, and then 400 μL of solution I [1% sarcosyl, 0.5 M NaCl, 1% sodium dodecyl sulfate (SDS)] was added. The tubes were kept at 37°C for 10 min with constant stirring (Agimaxx, Thermo Shaker) at 1,500 rpm and then kept for 1 h at 65°C with the same shaking. Then 400 μL phenol:chloroform:isoamyl alcohol 25:24:1 (PCI) was added and mixed by inverting, and centrifuged at 10,000 × *g* for 5 min at 37°C, followed by careful transfer of the supernatant to a new 1.5 mL reaction tube. Then, 10% of the total volume of sodium acetate (3 M, pH 5.2) and 60% isopropanol were added and mixed gently by inverting the tube 5 to 10 times. Then the tubes were centrifuged at 10,000 × *g* at 37°C for 5 min. The DNA was precipitated in the pellet and the liquid was removed. The pellet was washed with 1 mL of 70% ethanol and centrifuged at 10,000 × *g* for 3 min at 37°C. The supernatant was removed, and the DNA was resuspended in 20 μL TE 0.01X after drying.

PCR amplification was performed with the 18S SSU rRNA using primers forward NS1 (5′-GTAGTCATATGCTTGTCTC-3′) and reverse NS4 (5′-CTTCCGTCAATTCCTTTAAG-3′) (White et al., 1990), using the following PCR program: 94°C/3 min, followed by 35 cycles of 94°C/30 s, 56°C/45 s, 72°C/1 min and final extension of 72°C/10 min, 12°C/∞; 26S LSU rRNA (D1/D2 domains) using primers forward NL1 (5′-GCATATCAATAAGCGGAGGAAAAG-3′) and reverse NL4 (5′-GGTCCGTGTTTCAAGACGG-3′) (Kurtzman and Robnett, 1998) using the following PCR program: 96°C/3 min followed by 35 cycles at 96°C/30 s, 61°C/45 s, 72°C/1 min, 10°C/∞; and the ITS (Internal Transcribed Spacer) region containing primers forward ITS5 (5′-TCCGTAGGTGAACCTGCGG-3′) and reverse ITS4 (5′-TCCTCCGCTTATTGATATGC-3′) (White et al., 1990) using the following PCR program: 94°C/3 min followed by 35 cycles at 94°C/1 min, 55°C/1 min, 72°C/2 min, 10°C/∞. The final reaction volume of 15 μL contained: 8 μL PCRBIO HS Taq Mix (PCRBiosystems, London, UK), 0.5 μL of each primer (10 μM), 0.5 μL of 100% DMSO, 4.5 μL deionized H_2_O, and 1 μL DNA (10 ng/μL). Amplification products were subjected to agarose gel electrophoresis at 1% in 1X TBE buffer (10X: 108 g Tris-Base, 55 g boric acid, 8.3 g EDTA and 1 L deionized water, pH 8.3) and stained with GelRed^®^ (Biotium, Fremont, USA). Purification was performed using the GenElute PCR Clean-up Kit Sigma-Aldrich (St. Louis, MO, USA). The sequencing reaction was performed using 2.0 μL 5X buffer, 0.32 μL primer (10 μM), 0.3 μL BigDye^®^ 3.1 (Applied Biosystems, Waltham, USA), approximately 20 ng of purified DNA, and deionized H_2_O to achieve a total volume of 10 μL. The thermocycler program consisted of 28 cycles at 95°C for 15 s, followed by 50°C for 45 s and 60°C for 4 min, which were completed and maintained at 10°C. The sequencing reaction was purified according to the instructions in the BigDye^®^ manual (Applied Biosystems, Waltham, USA). Sequencing was performed using the 3500 Genetic Analyzer from Applied Biosystems, Waltham, USA. The sequences were edited in BioEdit 7.2.5 (Hall, 1999) and used to assemble the contigs. The contigs were used to search for homologous sequences in the National Center for Biotechnology Information (NCBI)–GenBank^1^ database.

### 2.5. Phylogenetic analyses

A phylogenetic analysis of the 26S LSU rRNA and a phylogeny of 18S were performed to include the isolated *Zygosaccharomyces* strains and other species previously described to cover most of the genus. After assembly of the contigs, sequences were manually aligned and trimmed in MEGA version X (Kumar et al., 2018). Phylogenetic and molecular evolutionary analyses were reconstructed using Bayesian inference (BI) in MrBAYES v.3.2.2 (Ronquist et al., 2012) with the substitution model general time-reversible with Gamma distribution for rate variation (GTR + I + G) for 26S phylogeny and GTR + I for 18S phylogeny. Two separate runs were performed, each consisting of a cold chain and three incrementally heated chains and a Markov Chain Monte Carlo (MCMC) with three million generations. One in each 50 generations were sampled to obtain Bayesian posterior probability (PP) values for the clades. Convergence occurred when the standard deviation (SD) of split frequencies fell below 0.01, and the first 10% of generations in the MCMC samples were discarded as burn-in. The final phylogenetic trees were visualized using iTOL v5 (Letunic and Bork, 2021). A maximum likelihood analysis was also conducted on the 26S LSU and 18S dataset using the GTR + I + G and TIM + I models, respectively. Runs were performed using PAUP 4.0^2^ software and probability bootstrap support values were generated from 1,000 replicates. The analysis of 26S LSU and the 18S included 47 and 36 nucleotide sequences, respectively. The first phylogenetic tree was rooted by *Saccharomyces cerevisiae* NRRL Y-12632 and the second one by *Wickerhamomyces anomalus* NRRL Y-366. The sequences are deposited in GenBank, and the deposit numbers are listed in Supplementary Table 1.

### 2.6. Ergosterol extraction

Microorganisms were grown in small Petri dishes (60 mm) containing 30 G agar medium for 15 days at 29°C. Cells were harvested with a loop and transferred to a 2 mL reaction tube (previously weighed) containing 1 mL of sterile saline (0.85% NaCl). This suspension was then vortexed for 10 s and centrifuged at 18,000 × *g* for 10 min. The supernatant was discarded, and the washing procedure was repeated twice. After the last cell wash centrifugation, the supernatant was discarded, and the reaction tube containing the cells was exposed to speed vacuum [Savant, Thermo Scientific (Waltham, MA, USA)] until the cells were completely dried. Each tube containing the samples was weighed and the weight of the empty tube was subtracted to obtain the final mass of each microorganism. For extraction, 250 μL of sterile saline was added to each reaction tube containing the fungal cell mass. This suspension was vortexed for 20 s, then 750 μl of chloroform was added. This mixture was vortexed for 20 s and then shaken at 1,500 rpm for 1 h at room temperature (Agimaxx, Thermo Shaker). The organic phase was collected and made up to a final volume of 1 mL with chloroform. The internal standard (β-sitosterol–25 μg/mL) was then added and analyzed by gas chromatography coupled to mass spectrometry (GC-MS). Analyses were performed at NPPNS-FCFRP-USP using a Shimadzu QP -2010 Plus gas chromatograph. A Phenomenex ZB-5MS (ZEBRON) column (30 m × 0.25 mm × 0.25 μm) with a flow rate of 1.50 mL/min, injection temperature of 250°C, interface of 300°C, and ionization source of 250°C in splitless mode was used. The temperature gradient used was 70°C for 4 min and then increased by 10°C/min to 300°C, remaining at 300°C for 15 min.

## 3. Results

### 3.1. Isolation and identification of fungi from stingless bees

Different nest sites of 19 species of SBs, distributed among 12 genera, were assessed for isolation of fungi, resulting in 35 fungal isolates. Thirty were obtained from brood cells, while four were isolated from honey and one from cerumen. All isolates had the 26S, 18S rRNA gene and ITS regions sequenced, resulting in 23 isolates belonging to the genus *Zygosaccharomyces*, seven were identified as *Monascus* spp., one as *Xerochrysium* sp. and one as *Leiothecium* sp (Supplementary Table 1).

Fungal filaments were observed and isolated from the brood cells of eight species of SBs distributed in four genera and eight species (*Scaptotrigona bipunctata*, *S. postica*, *S. tubiba*, *Tetragona clavipes*, *Melipona quadrifasciata*, *M. fasciculata*, *M. bicolor*, and *Partamona helleri*). All collected fungal filaments were identified as belonging to the genus *Zygosaccharomyces*, representing 42% incidence rate of *Zygosaccharomyces* sp. in brood cells of sampled SBs species. The *Zygosaccharomyces* isolates from the brood cells had the lowest query coverage and identity percentages (86–93%) with the closest described species from the Genbank database (*Z. rouxii*) and represent candidates for new species. *Zygosaccharomyces* were also isolated from honey of four species and from cerumen of one species (Supplementary Table 1). *Zygosaccharomyces osmophilus* was isolated from the honey of *T. clavipes*, *Geotrigona mombuca* and *M. quadrifasciata*, while *Z. siamensis* was isolated from honey and cerumen of the SB *Friesemelitta varia*. No fungal filaments were observed in the brood cells of 10 SBs species. Since it was not possible to visualize fungal filaments using a stereomicroscope, the cerumen was scraped off with a pointed spatula and the brood cells were washed with 30 G liquid medium for microbial isolation. This allowed the isolation of other fungal genera such as *Leiothecium*, *Xerochrysium*, and *Monascus*. Filamentous fungi such as *Monascus* sp., *M. ruber*, *Xerochrysium* sp. and *Leiothecium* sp. were found in colonies of *S. bipunctata*, *Tetragonisca angustula*, *Friesella schrottkyi*, *Nannotrigona testaceicornis*, and *M. quadrifasciata*. *Monascus ruber* (SBBCRP2) was isolated from the cerumen of the brood cell of *Scaptotrigona bipunctata*. These additional filamentous fungal isolates showed the lowest query coverage and identity percentages (82–85%) with species from the Genbank database and also represent candidates for new species.

**FIGURE 1:**
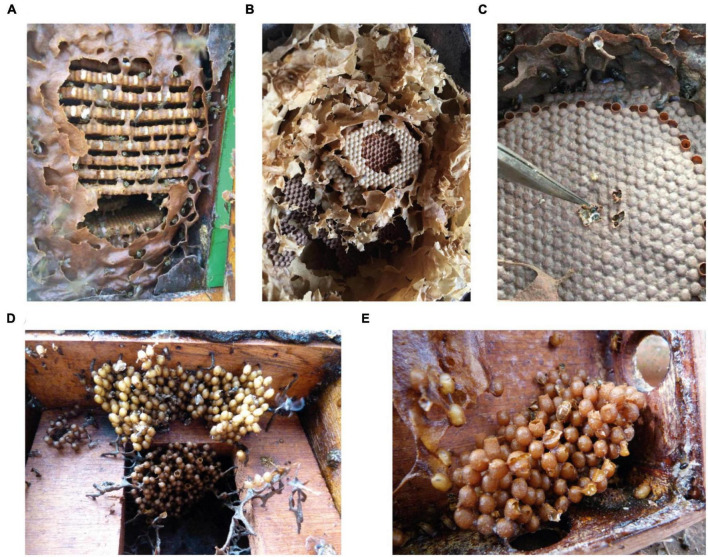
Brood cells of different stingless bee species. **(A)** *Tetragona clavipes* **(B)** *Nannotrigona testaceicornis* **(C)** *Scaptotrigona bipunctata* **(D)** *Frieseomelitta silvestrii* **(E)** *Friesemelitta varia*. The brood cells of **(A–C)** are arranged in the form of disks, while **(D,E)** are arranged in the form of clusters.

No fungal strain was isolated from the bees *Frieseomelitta silvestrii*, *F. doederleini*, *Leurotrigona muelleri*, *Melipona quinquefasciata*, and *Oxytrigona tataira*. Samples with low amounts of brood cells and the different larval stages could be limiting factors for the observation of *Zygosaccharomyces*, in addition to the difficulty of culturing this microorganism in less complex culture media. Fungal filaments were observed in SBs with disk-shaped brood cell structures (Figures 1A, C) and a larger diameter, but were not observed in other species with smaller diameters (Figures 1B, D, E) and cluster-shaped brood cells (Figures 1D, E). The size and shape of the brood cells may influence the visibility of the symbiont fungus.

**FIGURE 2:**
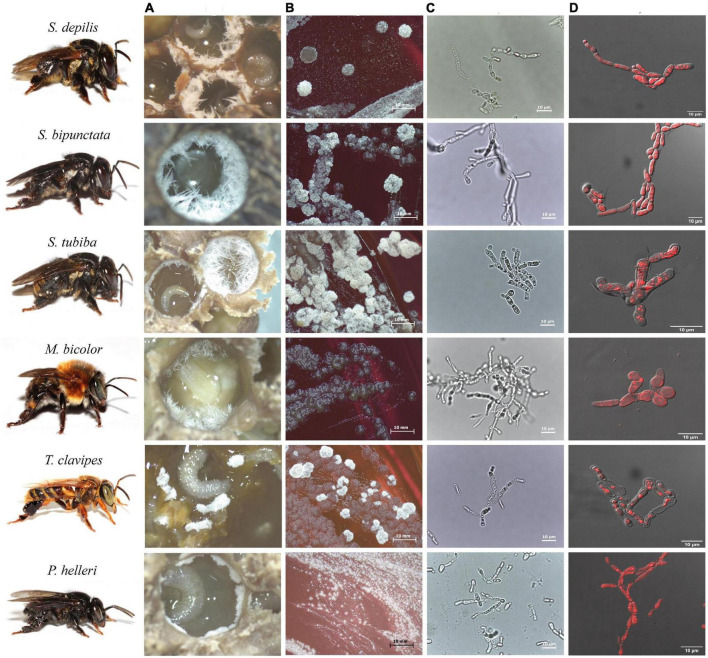
Images of *Zygosaccharomyces* spp. isolates from stingless bees *S. depilis*, *S. bipunctata*, *S. tubiba*, *M. bicolor*, *T. clavipes*, and *P. helleri*. **(A)** Fungal filaments present in the brood cells. **(B)** Microbial growth in Petri dish (14 days) **(C)** Optical microscopy images (100×). **(D)** Fluorescence microscopy images. Stingless bees’ photographs: credit Cristiano Menezes.

Fungal filaments are readily visible and occur in the early larval stages. In *Scaptotrigona* spp. and *Melipona bicolor*, *Zygosaccharomyces* isolates grow as long filaments and in substantial amounts, which facilitates isolation. The yeasts associated with *M. fasciculata*, *M. quadrifasciata*, and *P. helleri* form small filaments in small quantities in the first two SB species and in massive quantities in the last one. In contrast to the others, *Zygosaccharomyces* sp. from *T. clavipes* is found in clumps and also in large amounts (Figure 2A).

**FIGURE 3:**
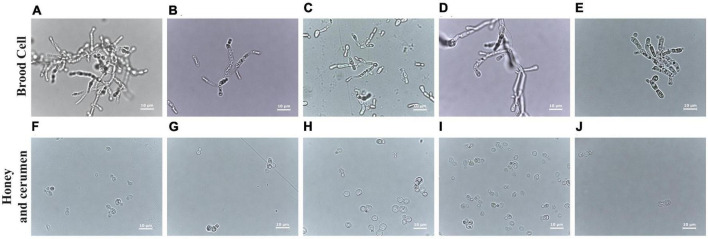
Comparative analysis of cells of *Zygosaccharomyces* spp. by optical microscopy at 100× magnification. Images **(A–E)** obtained from strains isolated from brood cells (**A:** *M. bicolor*; **B:** *T. clavipes*; **C:** *P. helleri*; **D:** *S. bipunctata* and **E:** *S. tubiba*). Strains **(F–I)** were isolated from other sources: **(F)** from cerumen of *F. varia*, and **(G–J)** from honey (**G:** *F. varia*, **H:** *G. mombuca*, **I:** *T. clavipes*, and **J:** *M. quadrifasciata*). The strains from the brood cells show pseudohyphae formation while strains from other parts of the colony present ovoid cells.

Cell morphologies of *Zygosaccharomyces* spp. varied according to the site of isolation. Isolates from brood cells form pseudohyphae in glucose-rich culture medium (30 G), while the others (honey and cerumen) have spherical and ovoid cells under the same growth conditions (Figure 3). The isolates that originated from different sites in the colonies (honey, pollen, refuse, and adult bees) grew faster during the treatment (at least two days) than the isolates that originated from brood cells (at least seven days), suggesting that there are physiological differences between them.

### 3.2. Ergosterol

*Zygosaccharomyces* strains from brood cells of different bees showed accumulation of intracellular lipids, as visualized by fluorescence microscopy (Figure 2D). GC-MS analyses of cell extracts of *Zygosaccharomyces* spp. confirmed the presence of ergosterol in all the samples (Supplementary Figure 1), as previously described for *Zygosaccharomyces* sp. isolated from *S. depilis* (Paludo et al., 2018).

### 3.3. Phylogenetic analyses

**FIGURE 4:**
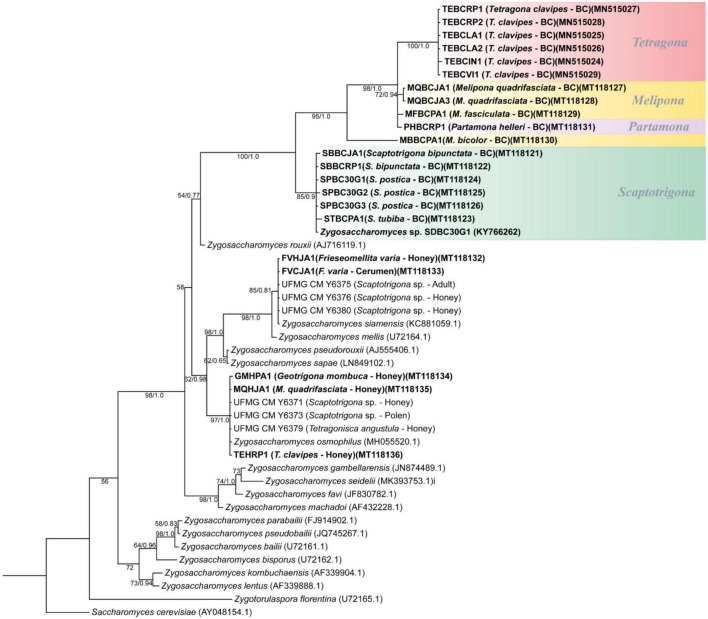
Phylogenetic tree from Bayesian analysis based on sequences of the D1/D2 domain of the 26S rRNA gene of *Zygosaccharomyces* spp. strains isolated from various stingless bee species (highlighted in bold) and from previously described *Zygosaccharomyces* species retrieved from GenBank. Strains isolated from brood cells are highlighted in color, grouped by stingless bee genera. Numbers on branches indicate PP/ML bootstrap values of support for each clade. A total of 540 aligned positions were analyzed. The scale bar represents 0.01 substitutions per nucleotide position. BC, brood cell.

Phylogenetic tree from Bayesian analyses using the sequences of the D1/D2 domain of the 26S gene was performed on *Zygosaccharomyces* spp. (Figure 4). The phylogeny obtained on D1/D2 sequences was confirmed by the phylogenies from the comparison of 18S genes (Supplementary Figure 2). The sequences used to construct the phylogenetic trees are deposited in GenBank and the 18S and 26S accession numbers are available in Supplementary Table 1. It was possible to observe the formation of a branch consisting only of *Zygosaccharomyces* spp. isolated exclusively from the brood cell of different species of SBs. The other *Zygosaccharomyces* species isolated from other nest sites are phylogenetically distant from this branch.

*Zygosaccharomyces* sp. SDBC30G1 was compared with *Z. rouxii* (the closest described species to the isolated brood cell branch), and a difference of 49 substitutions (8.3%) in D1/D2 sequences was observed in a dataset of 540 nucleotides (nt) (Supplementary Figure 3). In addition, the sequences of *Zygosaccharomyces* isolates from the brood cell of the other SBs species were compared with *Zygosaccharomyces* sp. SDBC30G1. The nt sequences of the SBs isolates from the genus *Scaptotrigona* showed an average percent identity of 99.5% in D1/D2 sequences reinforcing that they are the same species as *Zygosaccharomyces* sp. SDBC30G1. The sequences of isolates from the genus *Melipona* and the species *P. helleri* showed an average difference of 62 (11.5%) substitutions in D1/D2 sequences in a dataset of 540 nt. The same occurs with the *Zygosaccharomyces* isolates obtained from *T. clavipes*, which show an average difference of 76 (14.2%) substitutions in D1/D2 sequences in a dataset of 540 nt. These data support that we have candidates for new and distinct species of *Zygosaccharomyces* in the branch of brood cell isolates from different species of SBs.

## 4. Discussion

*Zygosaccharomyces* species have often been isolated from various parts of bee nests. In honey bees (*Apis mellifera*), different species of *Zygosaccharomyces* have been isolated from bee bread and honey (*Z. mellis*, *Z. rouxii*, *Z. siamensis*, *Z. favi*, and *Z. gambellarensis*) (Čadež et al., 2015; Rodríguez-Andrade et al., 2019), from pollen stored under warm temperatures (Friedle et al., 2021), and as part of the gut microbiota of these bees (Yun et al., 2018). *Zygosaccharomyces* is also present in various parts of SBs nests. Echeverrigaray et al. (2021) reported the presence of several species of *Zygosaccharomyces* in honey of SBs, such as *Z. mellis* (*M. quadrifasciata*, *Plebeia emerina*, *S. depilis*, and *Tetragonisca fiebrigi*), *Z. rouxii* (*P. droryana*, *P. emerina*, and *T. fiebrigi*), and *Z. siamensis* (*T. fiebrigi*). *Z. siamensis* has also been isolated from the honey of *Melipona interrupta* (Meireles et al., 2022). Haag et al. (2022) reported the presence of *Zygosaccharomyces* in the associated gut microbiota of *M. quadrifasciata* through a longitudinal metabarcoding study. Another species, *Zygosaccharomyces machadoi*, was isolated from garbage pellets of the SB *T. angustula* (Rosa and Lachance, 2005). Recently, a new species of *Zygosaccharomyces*, described as *Z. osmophilus*, was isolated from unripe and ripe honey and pollen of *S. bipunctata* and from ripe honey of *T. angustula* (Matos et al., 2020). These findings show the broad distribution of *Zygosaccharomyces* in distinct nest sites of bees. Similarly, *Zygosaccharomyces* species were also isolated from honey and cerumen of SBs in the present study. *Zygosaccharomyces osmophilus* was isolated from honey of *T. clavipes*, *G. mombuca*, and *M. quadrifasciata*, and *Z. siamensis* from honey and cerumen of SB *F. varia*.

The osmophilic yeast *Zygosaccharomyces* was first isolated from the brood cell of the SB *S. depilis*, and this led to the description of a novel symbiotic nutritive interaction involving this yeast and the larvae of *S. depilis* (Paludo et al., 2018). Larvae of *S. depilis* ingest *Zygosaccharomyces* sp. SDBC30G1, which grows inside the brood cells. *Zygosaccharomyces* sp. SDBC30G1 provides ergosterol to the larvae, and this steroid is a precursor for ecdysteroid biosynthesis leading to a proper metamorphic process. It has been suggested that this nutritional value is due to the cytoplasmic accumulation of lipid droplets (LPs) in *Zygosaccharomyces* sp. SDBC30G1 cells. In addition, this yeast forms pseudohyphae that could improve its availability to the larvae (Paludo et al., 2018).

This unprecedented association between *Zygosaccharomyces* sp. SDBC30G1 and *S. depilis*, prompted us to search for a similar insect-microbe interaction in other SBs species. Interestingly, in this work we describe the presence of *Zygosaccharomyces* spp. in the brood cells of several SBs species, such as *S. bipunctata*, *S. postica*, *S. tubiba*, *T. clavipes*, *P. helleri*, *M. quadrifasciata*, *M. bicolor*, and *M. fasciculata*.

Strains of *Zygosaccharomyces* spp. isolated from various SBs species accessed in this work form pseudohyphae, produce ergosterol and accumulate LPs intracellularly. This corroborates the possible nutritional function of these yeasts for SB larvae, as previously described for *S. depilis*.

While the isolates from the brood cells form pseudohyphae, the other isolates from different sites of the colony show round or ovoid cells under the same growth conditions. Some yeasts can have different forms, such as biofilm colonies, flocs and others, depending on their living conditions (Palková and Váchová, 2016). The cell morphology of yeasts in the genus *Zygosaccharomyces* is characterized by a predominance of spherical to ovoid and ellipsoidal cells. The formation of pseudohyphae is observed only in some species such as *Z. bailii*, *Z. lentus*, *Z. rouxii*, *Z. favi*, *Z. sapae*. In other species such as *Z. bisporus*, *Z. mellis*, *Z. kombuchaensis*, *Z. machadoi*, *Z. gambellarensis*, *Z. parabailii*, *Z. pseudobailii*, and *Z. seidelii*, the formation of pseudohyphae does not occur (Rosa and Lachance, 2005; James and Stratford, 2011; Torriani et al., 2011; Saksinchai et al., 2012; Solieri et al., 2013; Suh et al., 2013; Čadež et al., 2015; Brysch-Herzberg et al., 2020). Pseudohyphae formation can be triggered by low nitrogen levels and is a form of foraging (Cullen and Sprague, 2012). The location of these *Zygosaccharomyces* strains in the brood cells may be a determining factor in the expression of pseudohyphae. It is also likely that pseudohyphae are more easily ingested by the larvae since they are present on the surface of the larval food and have long filaments. This particular morphology of these yeasts (Figure 2) could favor the availability and uptake of nutrients for the larval development.

In yeast-insect associations the microorganism can be benefited by dispersal in the environment, protection from unfavorable conditions, and acquisition or transformation of specific nutrient sources (Stefanini, 2018). The brood cells of SBs are composed of cerumen and larval food, and their composition may vary in nature. Cerumen consists only of waxes and resins (Roubik, 2006), while larval food consists of water (40–60%), sugars (5–12%), and free amino acids (0.2–1.3%) (Hartfelder and Engels, 1989). These water-soluble components make up to 70% of the larval food composition. In addition, pollen and lipids are also present (Hartfelder and Engels, 1989). The low pH and low water availability of beebread in honey bees inhibit the growth of most microbes, favoring the growth of lactic acid bacteria and sugar-tolerant yeasts (Anderson and Mott, 2023). The yeasts in beebread are highly specialized commensal fungi able to neutralize the osmotic pressure due to the high sugar concentrations. Therefore, the host bees may evolutionarily select these yeasts to fulfill a nutritional function. Beebread in honey bees is similar to the brood cells in SBs, which represent a favorable environment for the growth and development of *Zygosaccharomyces* spp.

In addition, phylogenetic analyses showed similarities among *Zygosaccharomyces* isolates from the brood cells of analyzed SBs and *Zygosaccharomyces* sp. SDBC30G1, previously isolated from *S. depilis*, and differences among yeast strains from brood cells when compared with already known *Zygosaccharomyces* species from other colonies’ sites. Phylogenies showed a branch consisting of *Zygosaccharomyces* spp. isolated exclusively from the brood cells of different species of SBs. The other species of *Zygosaccharomyces* isolated from different nest sites are phylogenetically distant. The branch consists only of *Zygosaccharomyces* spp. strains isolated from the brood cells contain *Zygosaccharomyces* sp. SDBC30G1 associated with *S. depilis* (Paludo et al., 2018), and is divided into several groups. One group, which is quite clear and distinct from the others, consists of strains of *Zygosaccharomyces* spp. isolated from different species of the genus *Scaptotrigona*, suggesting that these strains belong to the same yeast species previously described for *S. depilis*. A second group consists of *Zygosaccharomyces* strains derived from the brood cells of several bee species of the genus *Melipona*, as well as the species *T. clavipes* and *P. helleri*. In contrast to *Scaptotrigona* isolates, *Zygosaccharomyces* spp. isolated from *Melipona* species probably belong to different species. In turn, *Zygosaccharomyces* isolates from different colonies of the species *T. clavipes* appear to belong to the same species, since they are closely related. The isolation of *Zygosaccharomyces* from different colonies of the same SB species, as in *T. clavipes*, is additional evidence that this microorganism is indeed present in this system and may have a specific function. The branch consisting only of isolates of *Zygosaccharomyces* spp. from the brood cells of different SBs species is present in both phylogenetic trees (Figure 4 and Supplementary Figure 2). These lineages are closely related, sharing a common ancestor, but included in a separate branch from other previously described *Zygosaccharomyces*. These analyses suggest that *Zygosaccharomyces* found in brood cells of SBs are specific to this system, despite differences in host insect morphology, behavior, and nest structure. This yeast, although derived from different bee species, could be associated with a specific nutritional function for bee larvae, as previously demonstrated for *S. depilis* (Paludo et al., 2018).

Other isolation techniques were used to isolate the microorganisms, such as scraping the cerumen from the brood cell or washing the entire brood cell with 30 G liquid medium, mainly in those species of SBs that did not have visible fungal filaments. This allowed the isolation of other species of non-*Zygosaccharomyces* fungi. Due to the use of this non-specific technique, it is not possible to conclude that these different isolated fungi have the same biological function as *Zygosaccharomyces* species for larval development of SBs.

*Monascus* SBBCRP2 isolated from the brood cell of *S. bipunctata* is morphologically similar to the strain of *Monascus ruber* SDBC1 previously isolated from the cerumen of the brood cell of *S. depilis* (Paludo et al., 2019). *Monascus* strains have been previously isolated from honey, pollen, and inside the nest of *Melipona scutellaris*, including *Monascus recifensis* (pollen), *M. ruber* (in the nest), *M. mellicola* (honey, pollen, and in the nest), *M. flavipigmentosus* (pollen and in the nest) (Barbosa et al., 2017), indicating a possible ecological interaction of this genus with SBs.

Altogether, our findings suggest a widespread symbiotic relationship between species of *Zygosaccharomyces* and SBs, and expand the current knowledge about the classical and non-classical mutualisms between fungi and insects (Biedermann and Vega, 2020). Meliponini are important pollinators of native plants and agricultural crops, playing important ecosystem services, and understanding their multipartite interactions can contribute to their management and preservation.

## Data availability statement

The datasets presented in this study can be found in online repositories. The names of the repository/repositories and accession number(s) can be found in the article/Supplementary material.

## Author contributions

GP, WM, CM, CP, CR, and MP performed conceptualization. GP, WM, and IC performed methodology. GP, WM, and MP performed data curation and wrote the original draft. GP, WM, IC, CM, CP, CR, and MP performed formal analysis, visualization, wrote, review and editing the manuscript. MP performed supervision. All authors contributed to the article and approved the submitted version.

## Footnotes

1
https://blast.ncbi.nlm.nih.gov/Blast.cgi

2
https://paup.phylosolutions.com/
