# Supplementary material for: Further evidences of an emerging stingless bee-yeast symbiosis
Source: Front Microbiol. 2023 Aug 11;14:1221724. doi: 10.3389/fmicb.2023.1221724 (PMC10450959; doi:10.3389/fmicb.2023.1221724)
Supplement: Supplementary file 1 [file Data_Sheet_1.docx]

Further evidences of an emerging stingless bee-yeast symbioses

Gabriela Toninato de Paula^1^, Weilan Gomes da Paixão Melo^2^, Ivan de Castro^3^, Cristiano Menezes^4^, Camila Raquel Paludo^5^, Carlos Augusto Rosa^6^, Mônica Tallarico Pupo^1*^

^1^Department of Pharmaceutical Sciences, School of Pharmaceutical Sciences of RibeirãoPreto, University of São Paulo, RibeirãoPreto, Brazil.

^2^Center for Agricultural and Natural Sciences and Letters, State University of the Tocantina Region of Maranhão, Estreito, Brazil.

^3^Department of Genetics, School of Medical Sciences of RibeirãoPreto, University of São Paulo, RibeirãoPreto, Brazil.

^4^Brazilian Agricultural Research Corporation, Embrapa Meio Ambiente, Jaguariúna, Brazil.

^5^Institute of Biological and Health Sciences, Federal University of MatoGrosso, Barra do Garças, Brazil.

^6^Departamento de Microbiologia, ICB, Universidade Federal de Minas Gerais, Belo Horizonte, Brazil.

*** Correspondence:**Mônica Tallarico Pupo
mtpupo@fcfrp.usp.br

Supplementary Material

Table S1. Data from strains of microorganisms isolated from different stingless bee nest sites.

Figure S1.Ergosterol analysis by GC-MS of *Zygosaccharomyces* spp. strains from different stingless bee species: (A) *Melipona bicolor,* (B) *Scaptotrigonabipunctata,* (C) *Meliponaquadrifasciata,* (D) *Tetragonaclavipes*. The images contain the chromatogram with the ergosterol retention time highlighted, followed by the identification suggested by the program and the ergosterol equivalent fragment ions*.*

**Figure S2.** Bayesian inference based on sequences of the 18S gene from *Zygosaccharomyces* spp. strains isolated from stingless bee species (highlighted in bold) and previously described *Zygosaccharomyces* species retrieved from GenBank. Strains isolated from brood cells are highlighted in color, grouped by stingless bee genera. Numbers on branches indicate PP/ML bootstrap values of support for each clade. A total of 914 aligned positions were analyzed. The scale bar represents 0.1 substitutions per nucleotide position. BC: Brood Cell.

Figure S3. Comparison of the 26S gene nucleotide sequences of the brood cell isolates of different SBs species, *Zygosaccharomyces* sp. SDBC30G1 and *Z. rouxii*. Sequences were aligned using MEGA version X (Kumar et al. 2018) and visualized in BioEdit 7.2.5 (Hall 1999).

Table S2. Data from strains of microorganisms isolated from different stingless bee nest sites.

| Stingless Bee Species | | Sample | Sample ID | GenBank Access Number (18S) | GenBank Access Number (26S) | Yeast Species | City, State, Country | GPS coordinates |
| --- | --- | --- | --- | --- | --- | --- | --- | --- |
| *Scaptotrigonabipunctata** | | Brood cell | SBBCJA1 | MT118313 | MT118121 | *Zygosaccharomyces* sp. | Jaguariúna-SP, Brazil | 22.732714 S 47.018359 W |
|  |  |  | SBBCRP1 | MT118312 | MT118122 | *Zygosaccharomyces* sp. | RibeirãoPreto-SP, Brazil | 21.169779 S 47.859670 W |
|  |  |  | SBBCRP2 | - | - | *Monascusruber* | RibeirãoPreto-SP, Brazil | 21.169779 S 47.859670 W |
| *Scaptotrigonapostica** | | Brood cell | SPBC30G1 | MT118315 | MT118124 | *Zygosaccharomyces* sp. | Belém-PA, Brazil | 1.436129 S 48.449181 W |
|  |  |  | SPBC30G2 | MT118316 | MT118125 | *Zygosaccharomyces* sp. | Belém-PA, Brazil | 1.436129 S 48.449181 W |
|  |  |  | SPBC30G3 | MT118317 | MT118126 | *Zygosaccharomyces* sp. | Belém-PA, Brazil | 1.436129 S 48.449181 W |
| *Scaptotrigonatubiba** | | Brood cell | STBCPA1 | MT118314 | MT118123 | *Zygosaccharomyces* sp. | Passos-MG, Brazil | 20.720259 S 46.613014 W |
| *Tetragoniscaangustula* | | Brood cell | TABCJA1 | - | - | *Xerochrysium*sp. | Jaguariúna-SP, Brazil | 22.732714 S 47.018359 W |
| *Tetragonaclavipes** | | Honey | TEHRP1 | MT118327 | MT118136 | *Zygosaccharomycesmellis* | RibeirãoPreto-SP, Brazil | 21.169779 S 47.859670 W |
|  |  | Brood cell | TEBCRP1 | MT118309 | MN515027 | *Zygosaccharomyces* sp. | RibeirãoPreto-SP, Brazil | 21.169779 S 47.859670 W |
|  |  |  | TEBCRP2 | MT118310 | MN515028 | *Zygosaccharomyces* sp. | RibeirãoPreto-SP, Brazil | 21.169779 S 47.859670 W |
|  |  |  | TEBCLA1 | MT118307 | MN515025 | *Zygosaccharomyces* sp. | Luís Antonio-SP, Brazil | 21.575217 S 47.746817 W |
|  |  |  | TEBCLA2 | MT118308 | MN515026 | *Zygosaccharomyces* sp. | Luís Antonio-SP, Brazil | 21.575217 S 47.746817 W |
|  |  |  | TEBCIN1 | MT118306 | MN515024 | *Zygosaccharomyces* sp. | Indaiatuba-SP, Brazil | 23.066368 S 47.170355 W |
|  |  |  | TEBCVI1 | MT118311 | MN515029 | *Zygosaccharomyces* sp. | Viçosa-MG, Brazil | 20.758624 S 42.868174 W |
| *Frieseomelittavaria* | | Honey | FVHJA1 | MT118324 | MT118132 | *Zygosaccharomycessiamensis* | Jaguariúna-SP, Brazil | 22.732714 S 47.018359 W |
|  |  | Cerumen | FVCJA1 | MT118325 | MT118133 | *Zygosaccharomycessiamensis* | Jaguariúna-SP, Brazil | 22.732714 S 47.018359 W |
|  |  | Brood cell | FVBCRP1 | NA | NA |  | RibeirãoPreto-SP, Brazil | 21.169779 S 47.859670 W |
|  |  |  | FVBCRP2 | NA | NA |  | RibeirãoPreto-SP, Brazil | 21.169779 S 47.859670 W |
| *Frieseomelittasilvestrii* | | Brood cell | NI |  |  |  | RibeirãoPreto-SP, Brazil | 21.169779 S 47.859670 W |
| *Frieseomelittadoederleini* | | Brood cell | FDBCPA1 | NA | NA |  | Passos-MG, Brazil | 20.720259 S 46.613014 W |
| *Friesellaschrottkvi* | | Brood cell | FSBCJA1 | - | - | *Leiothecium*sp. | Jaguariúna-SP, Brazil | 22.732714 S 47.018359 W |
|  |  |  | FSBCJA2 | - | - | *Monascus*sp. | Jaguariúna-SP, Brazil | 22.732714 S 47.018359 W |
| *Geotrigonamombuca* | | Honey | GMHPA1 | MT118326 | MT118134 | *Zygosaccharomycesmellis* | Passos-MG, Brazil | 20.720259 S 46.613014 W |
| *Leurotrigonamuelleri* | | Brood cell | NI |  |  |  | Jaguariúna-SP, Brazil | 22.732714 S 47.018359 W |
| *Nannotrigonatestaceicornis* | | Brood cell | NTBCJA1 | - | - | *Monascus*sp. | Jaguariúna-SP, Brazil | 22.732714 S 47.018359 W |
|  |  |  | NTBCJA2 | - | - | *Monascus*sp. | Jaguariúna-SP, Brazil | 22.732714 S 47.018359 W |
|  |  |  | NTBCRP1 | - | - | *Monascus*sp. | RibeirãoPreto-SP, Brazil | 21.169779 S 47.859670 W |
| *Plebeiadroryana* | | Brood cell | PDBCJA1 | - | - | *Monascus*sp. | Jaguariúna-SP, Brazil | 22.732714 S 47.018359 W |
| *Meliponaquadrifasciata** | | Honey | MQHJA1 | - | MT118135 | *Zygosaccharomycesmellis* | Jaguariúna-SP, Brazil | 22.732714 S 47.018359 W |
|  |  | Brood cell | MQBCJA1 | MT118318 | MT118127 | *Zygosaccharomyces* sp. | Jaguariúna-SP, Brazil | 22.732714 S 47.018359 W |
|  |  |  | MQBCJA2 | - | - | *Monascus*sp. | Jaguariúna-SP, Brazil | 22.732714 S 47.018359 W |
|  |  |  | MQBCJA3 | MT118319 | MT118128 | *Zygosaccharomyces* sp. | Jaguariúna-SP, Brazil | 22.732714 S 47.018359 W |
|  |  |  | MQBCPA1 | - | - | *Zygosaccharomyces* sp. | Passos-MG, Brazil | 20.720259 S 46.613014 W |
| *Meliponaquinquefasciata* | | Brood cell | NI |  |  |  | Passos-MG, Brazil | 20.720259 S 46.613014 W |
| *Melipona fasciculata** | | Brood cell | MFBCPA1 | MT118321 | MT118129 | *Zygosaccharomyces* sp. | Passos-MG, Brazil | 20.720259 S 46.613014 W |
| *Melipona bicolor** | | Brood cell | MBBCPA1 | MT118322 | MT118130 | *Zygosaccharomyces* sp. | Passos-MG, Brazil | 20.720259 S 46.613014 W |
| *Oxytrigonatataira* | | Brood cell | NI |  |  |  | Passos-MG, Brazil | 20.720259 S 46.613014 W |
| *Partamonahelleri** | | Brood cell | PHBCRP1 | MT118323 | MT118131 | *Zygosaccharomyces* sp. | RibeirãoPreto-SP, Brazil | 21.169779 S 47.859670 W |
|  | (*)Fungal filaments were observed in the brood cell. (NI) No isolation. (NA) No amplification of 26S and/or 18S. (-) Sequence not deposited in GenBank. | | | | | | | |
