# Supplementary figures and images for: Further evidences of an emerging stingless bee-yeast symbiosis
Source: Front Microbiol. 2023 Aug 11;14:1221724. doi: 10.3389/fmicb.2023.1221724 (PMC10450959; doi:10.3389/fmicb.2023.1221724)

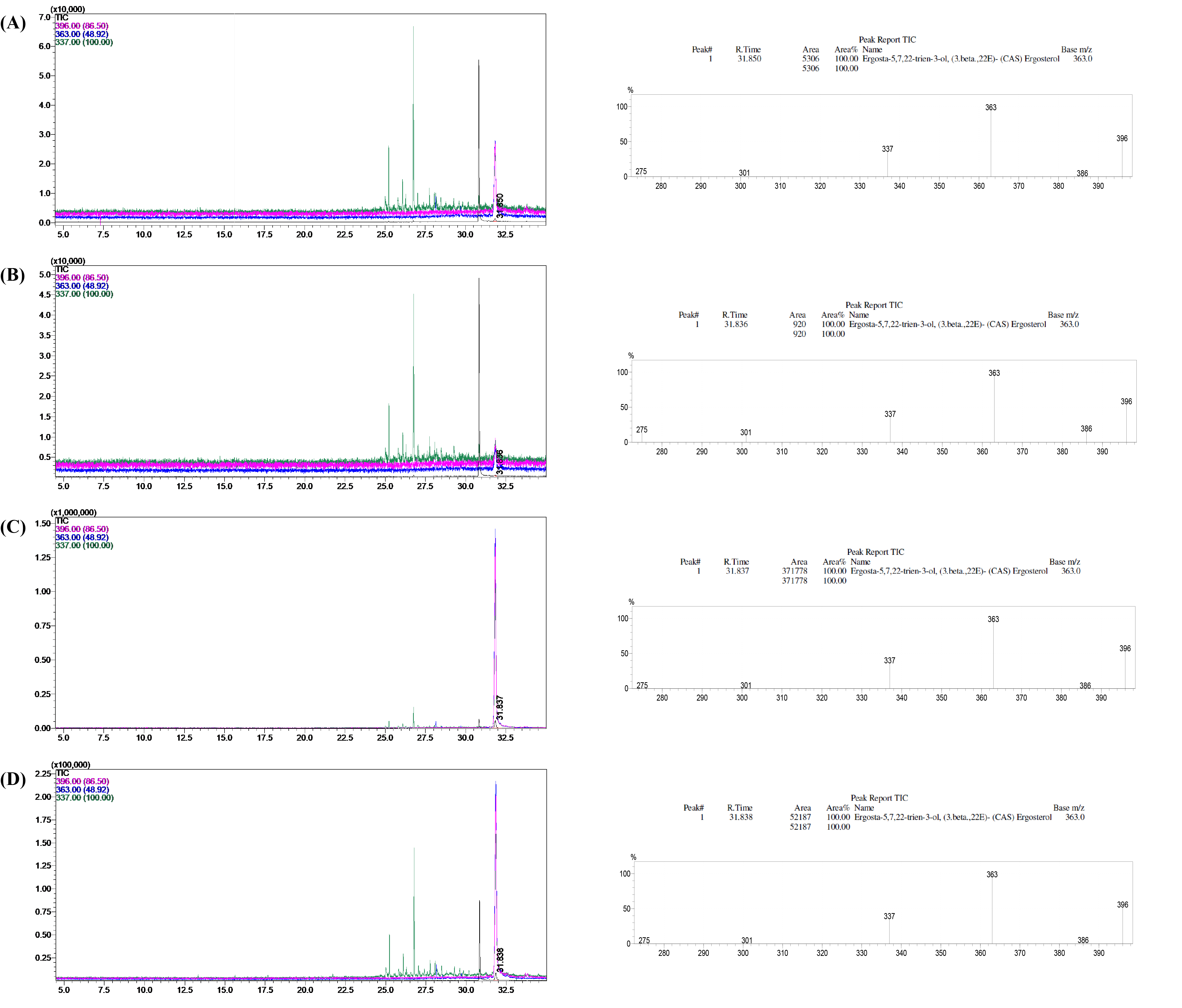

Supplement: Supplementary file 2 [file Image_1.JPEG]

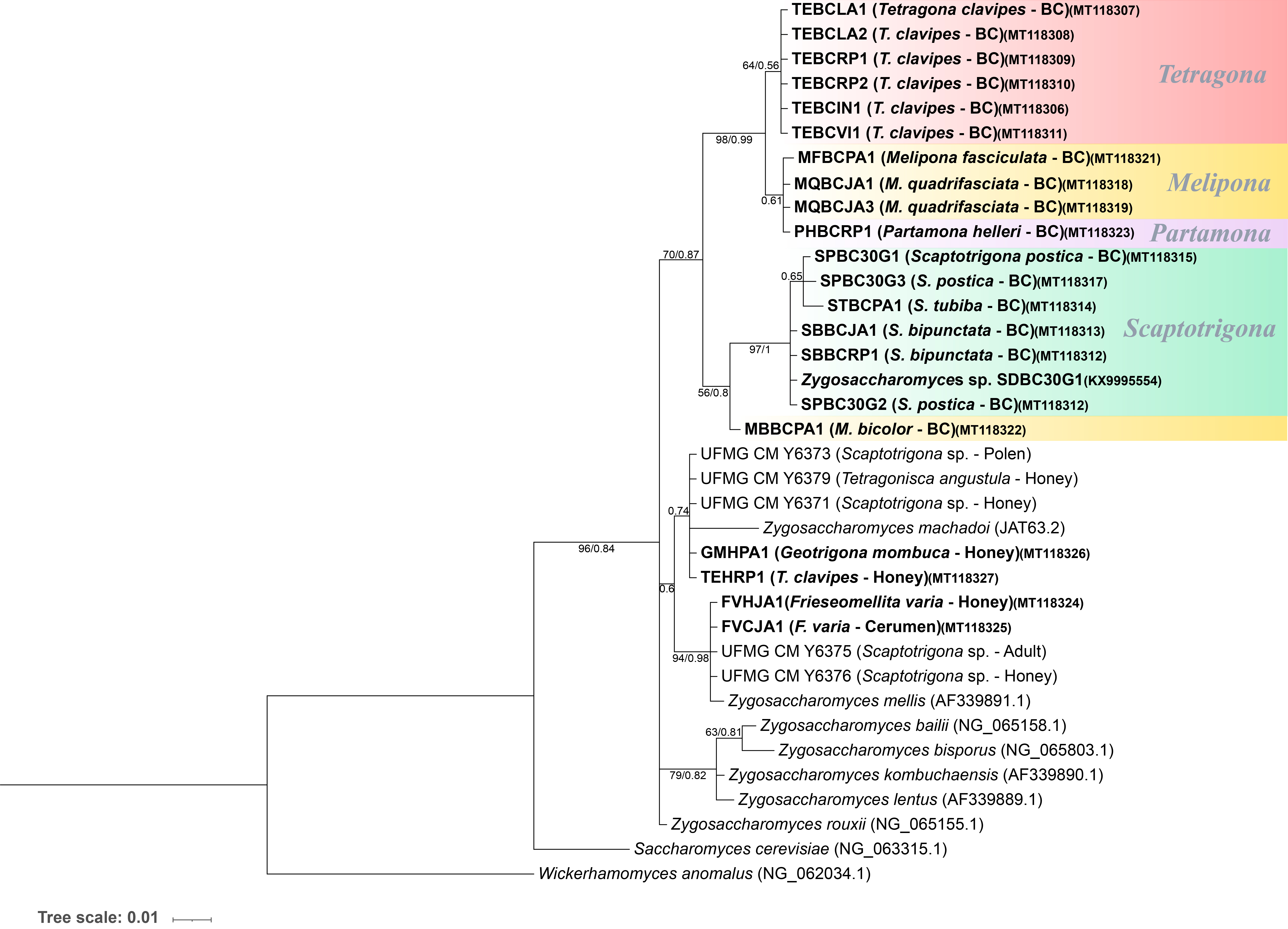

Supplement: Supplementary file 3 [file Image_2.JPEG]

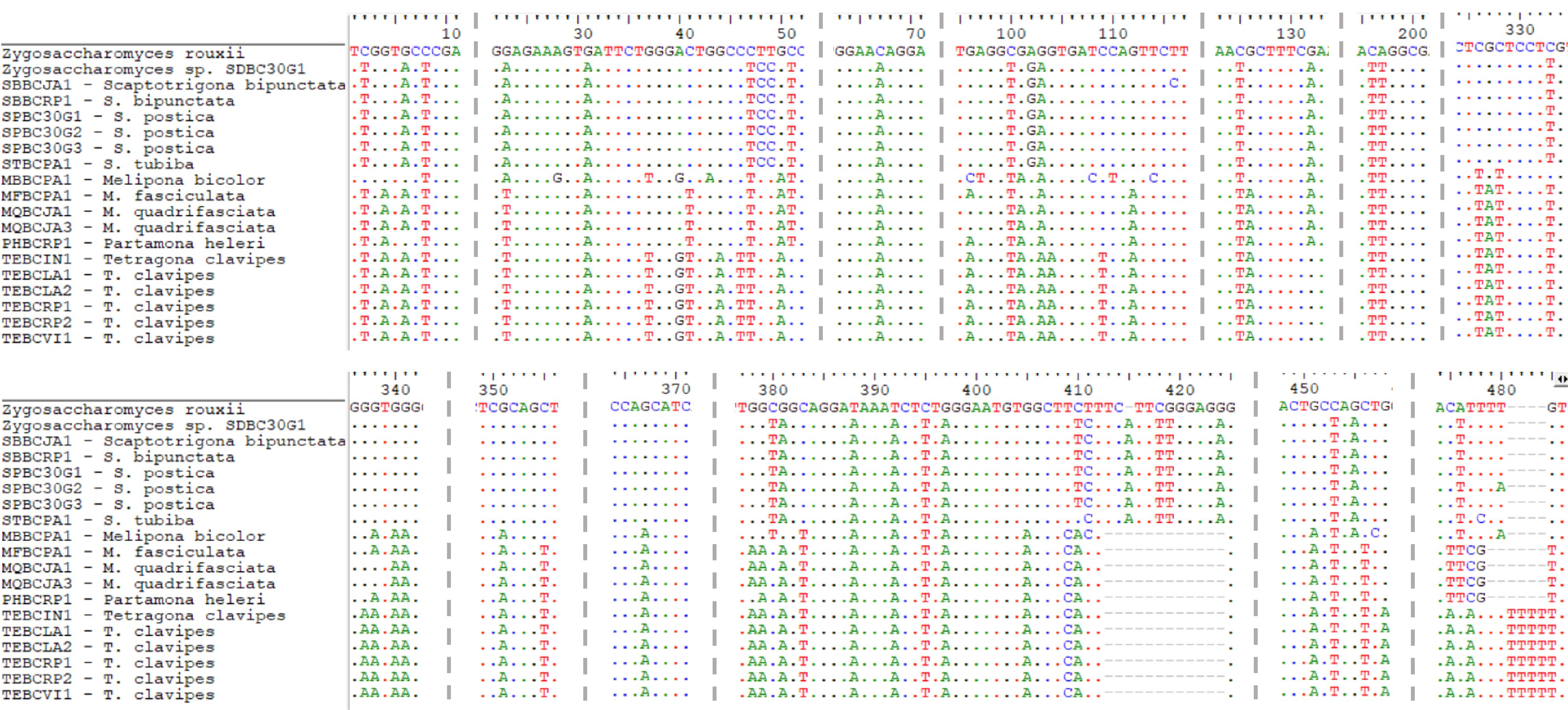

Supplement: Supplementary file 4 [file Image_3.JPEG]
